# Beyond fludarabine: pentostatin plus cyclophosphamide are a well-tolerated alternative in reduced intensity conditioning

**DOI:** 10.1038/s41409-022-01819-y

**Published:** 2022-09-17

**Authors:** Dimana Dimitrova, Jennifer A. Kanakry

**Affiliations:** Center for Immuno-Oncology, Center for Cancer Research, National Cancer Institute, National lnstitutes of Health, Bethesda, MD USA

**Keywords:** Phase II trials, Immunological disorders, Haematological diseases, Haematological cancer, Stem-cell research

## To the Editor:

The fludarabine shortage is causing unease within the hematopoietic cell transplantation (HCT) and cell therapy community as most reduced intensity (RIC) platforms along with conditioning for engineered T cell products rely on fludarabine for lymphodepletion. A recent national survey drew attention to the significant impact of oncology drug shortages causing treatment delays and rising hospital costs [1]. Due to manufacturing delays, access to fludarabine specifically has been disproportionately affected in 2022. In this correspondence, we aim to highlight the fludarabine-free RIC regimen that our group has used since 2015 as the backbone for 89 allogeneic RIC HCTs to date, for malignant and nonmalignant indications, in the context of clinical trials at the National Institutes of Health. In 2011, Mariotti et al. demonstrated equivalent T lymphodepletion by pentostatin and low-dose, hyperfractionated cyclophosphamide (PC) as compared to fludarabine plus low-dose cyclophosphamide, but increased functional immunosuppression by pentostatin in a murine model [2]. These findings were translated to the bedside in nonmyeloablative HCT platforms, where effective T lymphodepletion was again demonstrated [3]. In 2015, a novel, RIC allogeneic HCT platform combined a PC backbone with 2 days of pharmacokinetically-dosed busulfan (Bu2), T cell replete grafts, and post-transplantation cyclophosphamide-based graft-versus-host disease (GVHD) prophylaxis (clinicaltrials.org identifier NCT02579967). The intentions in designing the conditioning regimen were to intensify lymphodepletion by using pentostatin instead of fludarabine, while also avoiding toxicities incurred by total body irradiation and myeloablative doses of alkylating agents. The first 20 patients were described by Dimitrova et al. and included children and adults with various inborn errors of immunity, of whom 30% also had a hematologic malignancy [4, 5]. Results were remarkable for 1-year overall survival of 90%, low incidence of organ toxicity and acute GVHD but even more so the absence of any chronic GVHD, while 1-year graft failure incidence of 10% was comparable to fludarabine-containing RIC regimens [6, 7]. In approaches branching off from our original platform but using the same PC-Bu2 backbone, immunosuppression duration has been reduced with good success (*n* = 23 further RIC HCTs, NCT02579967) [8], and distal serotherapy with equine anti-thymocyte globulin added for enhanced lymphodepletion in patients with underlying T cell dysregulation (*n* = 17, NCT03663933) and/or T cell lymphoma (*n* = 29, NCT03922724) [9, 10], Table 1. We anticipate the regimen may also be appropriate for patients with myeloid disorders who cannot tolerate a myeloablative HCT, given early full donor myeloid chimerism in the majority of recipients. One drawback to our regimen is that the conditioning is longer than typical fludarabine-based regimens. However, it is also very well-tolerated and can be administered in the outpatient setting to avoid overly lengthy inpatient hospitalization. While other alternatives exist, such as clofarabine, pentostatin is associated with fewer infections, less nephrotoxicity, and less hepatotoxicity, and is less costly than clofarabine [11–13]. Thus, with fludarabine difficult to obtain or unavailable, our platform offers an excellent alternative that may prevent treatment delays for patients in need of RIC HCT while allowing fludarabine to be preserved for other oncologic uses.

**Table 1:** HCT platform details and HCT experience to date on 3 clinical trials using PC-Bu2 conditioning backbone.

Characteristic (*n* HCTs to date)	NCT02579967 (*n* = 43)	NCT03663933 (*n* = 17)	NCT03922724 (*n* = 29)
Recipient age	Adult ≤75 years (*n* = 32) Pediatric ≥4 years (*n* = 11)	Adult (*n* = 12) Pediatric ≥4 years (*n* = 5)	Adult (*n* = 28) Pediatric ≥12 years (*n* = 1)
Diagnosis	IEI	T cell dysregulation disorders, including IEI, aplastic anemia with LGL, T cell CAEBV	Relapsed/refractory peripheral T cell lymphoma
Conditioning	Pentostatin 4 mg/m^2^ on days −11 and −7 Cyclophosphamide 5 mg/kg/day on days −11 to −4 Busulfan Target daily AUC 4 600 µmol-min on days −3 and −2	Equine ATG 40 mg/kg on days −14 and −13 Pentostatin 4 mg/m^2^ on days −11 and −7 Cyclophosphamide 5 mg/kg/day on days −11 to −4 Busulfan Target daily AUC 4 600 µmol-min on days −3 and −2	Equine ATG 40 mg/kg on days −14 and −13 Pentostatin 4 mg/m^2^ on days −11 and −7 Cyclophosphamide 5 mg/kg/day on days −11 to −4 Busulfan Target daily AUC 4 600 µmol-min on days −3 and −2
Donor	Matched related (*n* = 9) Haploidentical (*n* = 11) Matched unrelated (*n* = 20) Mismatched unrelated (*n* = 3)	Matched related (*n* = 1) Haploidentical (*n* = 5) Matched unrelated (*n* = 9) Mismatched unrelated (*n* = 2)	Matched related (*n* = 3) Haploidentical (*n* = 17) Matched unrelated (*n* = 6) Mismatched unrelated (*n* = 3)
Graft	T-replete, marrow (*n* = 33) preferred over PBSC (*n* = 10)	T-replete PBSC (*n* = 17)	T-replete PBSC (*n* = 29)
GVHD prophylaxis	Post-transplantation cyclophosphamide 50 mg/kg/day on days +3 and +4 Sirolimus day +5 to +90 (originally to +180) +/− Mycophenolate mofetil day +5 to +18 (originally to +35)	Post-transplantation cyclophosphamide 50 mg/kg/day on days +3 and +4 Mycophenolate mofetil day +5 to +25 Tacrolimus day +5 to +90	Post-transplantation cyclophosphamide 50 mg/kg/day on days +3 and +4 Mycophenolate mofetil day +5 to +25 Sirolimus day +5 to +60

*ATG* anti-thymocyte globulin, *AUC* area under the curve, *CAEBV* Chronic Active EBV, *EBV* Epstein-Barr virus, *IEI* inborn errors of immunity, *LGL* large granular lymphocytosis, *PBSC* peripheral blood stem cells.
